# A Structural Equation Model (SEM) for the socio-economic impacts of ecotourism development in Malaysia

**DOI:** 10.1371/journal.pone.0273294

**Published:** 2022-08-29

**Authors:** Md. Abud Darda, Md. Anowar Hossain Bhuiyan

**Affiliations:** 1 Natural Science Group, National University Bangladesh, Gazipur, Bangladesh; 2 Business Studies Group, National University Bangladesh, Gazipur, Bangladesh; Fiji National University, FIJI

## Abstract

The present study investigates the perception of local communities and obtains the socio-economic impacts of ecotourism development in Terengganu, Malaysia. Two ecotourism places of Terengganu namely Lake Kenyir and Sekayu Recreational Forest had purposively been chosen for study. A non-probability convenience sampling was adopted for sample selection and a structured questionnaire was administered among 310 respondents to investigate the perception of the local communities. Factor analysis was done to identify the latent constructs and a theoretical Structural Equation Model (SEM) was proposed and tested. Results show that employment opportunities, homestay accommodations, and community participation are some positive socio-economic impacts of ecotourism development. Moreover, degradation of natural resources and break-up of religious traditions have been identified as negative socio-economic impacts. To ensure sustainable ecotourism development and endure the maximum benefit to the local communities, these negative impacts should be minimized. Encouragement should be given to local communities to accelerate the positive impacts of ecotourism.

## Introduction

Tourism impacts on local communities may be analyzed in several aspects such as social, economic, cultural, and environmental features. Tourism provides several positive impacts such as creating economic growth, increasing the standard of living, infrastructure development, and more opportunities for business activities and investment [1, 2]. Canavan [3] revealed through a study that local peoples have appreciated the social benefits of tourism activities and paid more attention to increasing the number of tourists. Sanchez Canizares [4] identified the tourists’ and hosts’ perceptions of tourism development on the African Island. Their study showed that tourists were satisfied with the services provided by the hosts and local communities expected support from tourists in tourism development for ensuring local benefits. Moreover, Local communities’ positive attitude toward tourism development depends on the positive impacts of tourism and social relations and communication between residents and tourists [1, 2].

Ecotourism is recognized as a conservation tool for natural resources and biodiversity of an area with consideration of poverty alleviation and rural development [5]. This tourism segment involves the local communities in tourism activities and operates based on local cultural and natural assets [6]. Jeffrey [7] identified through a study in the Philippines that sustainable ecotourism development ensures more income and job opportunities for the locals, has a positive impact on the environment, and offers a higher price for goods. Again, ecotourism can able to combine people from diverse cultures, ethnicities, lifestyles, and socioeconomic conditions [8]. Moreover, ecotourism development ensures well-being for the local communities, such as maximizing the benefits for locals [5, 9], creating opportunities in tourism planning [10], taking part in decision-making [11], environmental advantages [12], conservation of flora and fauna [13], economic benefits [14], environmental conservation [15], employment opportunities [16, 17], improve the livelihoods [18], improve and diverse local economy [19], support homestay accommodations [15, 17], provision of business opportunities and improve family income [20]. However, local communities were dissatisfied with the negative effects of tourism development such as illegal activities, crime, shortage of goods and services, crowding, and congestion [1, 4]. Moreover, ecotourism creates some problems for local communities such as environmental problems [21], and influences change in religious beliefs [22].

Several studies revealed that ecotourism development generates positive socio-economic impacts on the local communities in Malaysia. Mohamad and Hamzah [23] showed that local communities’ social, economic, and environmental concerns are important for tourism development in any area. Through a study in Sabah, they revealed that local communities have willing to participate in the decision-making and income distribution from tourism development. Bhuiyan et al. [24] showed that ecotourism development provides social, economic, and environmental benefits for the local communities in Sekayu Recreational Forest. Ismail et al. [25] identified that local communities express their positive views towards tourism development in small islands of Malaysia due to their positive socio-economic benefits. Moreover, some researchers also pointed out some negative socio-economic impacts due to improper ecotourism development in Malaysia. Shuib et al. [26] identified that the residents reported illegal activities such as drug marketing and theft cases due to tourism development in Tioman, Malaysia. Ecotourism development did not provide employment opportunities to the local people of the Lagong Hill Forest Reserve in Malaysia due to limited access and awareness [27]. Daldeniz and Hampton [28] identified some reasons for the negative perception of local communities towards tourism development in Malaysia such as environmental pollution, lack of employment opportunities, limited access to tourism business, cultural degradation, and less community participation.

Terengganu is one of the states on the East Coast of Peninsular Malaysia. This state is blessed with natural resources, unspoiled beauties, islands, beaches, Islamic heritage, and cultural attractions. Moreover, this state has a lush tropical landscape and nature, marine parks, and a wide variety of trees and food crops. The people of this state are friendly and welcoming hosts. All of these are suitable for potential ecotourism development in this state [29]. Some socio-economic backwardness remains in Terengganu state compared to the other states of Malaysia. A lower level of income, unemployment, poverty, less urbanization, less investment, and poor infrastructure development are mentionable among them. The Malaysian government emphasized ecotourism development in this state based on tourism-friendly attractions to improve socio-economic backwardness as well as local communities’ well-being [30].

The previous tourism studies on Terengganu concentrated on the local communities’ perspectives on ecotourism development in this state. Mansor et al. [31] emphasized local business, promoting local products, and local employment for tourism development in Terengganu. Afthanorhan et al. [32] have identified communities’ perceptions of tourism development in Terengganu. Their study revealed that communities have positive perceptions of economic benefits, business opportunities, and employment creation due to tourism development. Again, the local communities expressed their negative views towards the degradation of the environment and natural resources due to tourism development [32]. The study of Adam et al. [33] on Lake Kenyir indicated that local communities influence ecotourism development considering several impacts such as income generation, participation in ecotourism development, environmental aspects, spiritual life, and community well-being. Through a study on Lake Kenyir, Yusof et al. [34] identified that sustainable use of natural resources and respect for local culture and environment are necessary for ecotourism development in Terengganu. Osnin and Rahman [35] pointed out that tourism activities in Lake Kenyir must consider an economic contribution and a safe environment to ensure the well-being of local communities.

The success of ecotourism development is depending on ensuring the benefits gathering for the local communities through tourism activities. The local communities are willing to gather their social and economic well-being from the ecotourism development. The previous studies on Malaysia give emphasize several economic benefits of local communities from ecotourism like job opportunities [32, 36], local accommodation [37, 38], proper utilization of natural resources [24, 39], improve living quality [24], promote local goods [31], and create local business opportunity [40]. Moreover, several previous studies on Malaysia also emphasize on social benefits of local communities from ecotourism such as reducing negative impacts [32], preserving cultural values and tradition [41], respecting the religious tradition [33], participation in decision making [28], and community well-being [35]. Thus, it is pointed out that socio-economic impact assessment on local communities is essential for ecotourism development in Malaysia. Because, successful ecotourism development is ensuring positive socio-economic impacts on local communities such as economic benefits, social enhancement, and environmental well-being.

The present study investigates the perception of local communities and tries to identify the socio-economic impacts of ecotourism development in Terengganu state in Malaysia. The research questions addressed in this study can be outlined as,

- How does ecotourism development impacts the local communities?
- What are the perceived economic impacts of ecotourism development?
- What is the social impact of ecotourism experienced by the local communities?

## Literature review

Ecotourism is a form of nature-oriented tourism, contributing to social, economic, and environmental benefits in a way of scientific and public awareness in the 1990s [42]. The main components of ecotourism are gathering experience regarding natural attractions, local culture and traditions, maintaining sustainability, ensuring educational and interpretive facilities for tourists, managing the active involvement of local people and ensuring their well-being, preserving the conservation, and protecting the environment from degradation [43]. Amoamo et al. [14] pointed out that ecotourism can contribute both positively and negatively to the livelihood of local communities in terms of social, economic, and environmental aspects.

Kumar et al. [44] addressed that ecotourism creates economic opportunities and preserves the cultural integrity of the local people that sustain their livelihood. Their study on Jim Corbett National Park in Uttarakhand, India revealed that ecotourism provides support for the livelihood of local communities. Amalu et al. [45] addressed that ecotourism provides benefits for locals such as jobs and business opportunities, increases family income, and promotes local products. Based on a study in Ethiopia, Asfaw [46] argued that ecotourism improves the livelihood of local communities through preservation and management of natural resources, diversification of livelihood, and poverty reduction. Yanes et al. [10] pointed out that community participation in the decision-making of ecotourism can ensure economic benefits distribution among the residents.

Latip et al. [47] found that social, economic, and environmental benefits are necessary to ensure community support for tourism development in Malaysia. The study of Bhuiyan et al. [24] recommended some initiatives for ecotourism development in Terengganu such as conservation of natural resources, improving local people living quality, sustainable harvest, and keeping the environment free from pollution. Jaafar [37] highlighted homestay accommodation as a suitable business for tourism development in Malaysia. Shuib et al. [26] identified that tourism development is helpful for employment opportunities, increases the price of goods, creates income generation, and promotes local handicrafts and agriculture products in Malaysia. Ecotourism development can increase non-agricultural employment in sales and services due to tourism activities in Malaysia. The residents can operate small businesses for food and handicraft selling to attract tourists [40].

Saikim et al. [39] argued that sustainable ecotourism should be ensured through the participation of local communities in the management of conservation activities and control of revenue generation. A study on local communities in Sabah revealed that if tourism revenue declines, the communities decrease their cooperation toward ecotourism activities [39]. Jaafar et al. [36] identified that local communities in Malaysia achieved benefits from ecotourism development such as income generation, improved living conditions, business opportunities, employment creation, etc. The local communities at Kinabalu National Park in Sabah showed their negative perception due to uncontrolled ecotourism development [36]. The study on marine protected areas in Malaysia showed that local communities emphasize several attributes for ecotourism development such as conserving natural resources, economic benefits, participation in tourism development, and preserving the cultural tradition [41]. The local communities prioritized homestay accommodation for ensuring benefits through ecotourism development in Malaysia. Homestay accommodation can positively contribute to local livelihoods, income generation, and participation opportunities in tourism development [38]. Table 1 highlights the possible socio-economic impacts on local communities due to ecotourism development in Malaysia.

**Table 1:** Socio-economic impacts of ecotourism development.

Socio-economic Impacts	References
Prices of goods	[26, 36]
Employment opportunity	[32, 36]
Homestay accommodation	[37, 38]
Overall negative impact	[28, 32]
Preserve cultural tradition	[34, 41]
Respectful to religious tradition	[33]
Degradation of natural resources	[24, 39]
Participation in tourism development	[23, 41]

## Materials and methods

### Study sites

According to the East Coast Economic Region (ECER) Master Plan of Malaysia, the Lake Kenyir and the Sekayu Recreational Forest of Terengganu have been identified as prospective ecotourism sites for their natural beauties, recreational facilities, and tourism activities. Both the ecotourism sites are situated in Hulu Terengganu district which is staying comparatively below the socio-economic standard of the Terengganu state level as well as the national level, Malaysia. For this reason, ecotourism activities should be enhanced in this district for increasing the socio-economic well-being of local communities [29]. The present study has chosen these two ecotourism attractions as study sites.

Sekayu is the largest and a popular recreational forest in Terengganu which provides a natural and educational experience, local culture, and environmental attractions for tourists. Sekayu Recreational Forest was established in 1974 and was officially launched in 1985. It is located within the Hulu Terengganu forest reserve in Terengganu. There is a fruit orchard, a mini zoo, a bird park, and a flower garden in the forest area [48].

Lake Kenyir is the largest man-made lake in Malaysia and consists of forest reserves, forest recreation areas, mountain ranges, wildlife, and natural landscapes. It is situated in the eastern part of Terengganu sharing its border with Kelantan in the west and Pahang in the south. The total area of the lake is 260,000 hectares and it is one of the gateways to the National Park. The lake area covers 340 islands including hilltops and highlands, more than 14 waterfalls, numerous rapids, and rivers [49].

### Sample

The study follows a non-probability convenience sampling technique for the sample selection. This cheapest and easiest technique of sample selection gives researchers the freedom to choose the sample from a large universe. Willson and Mclntosh [50] used this design to determine the sample from tourists and measure the perception of cultural and heritage attractions in the USA. Bhuiyan and Darda [51] used this sample design to measure the tourists’ satisfaction in Lake Kenyir, Malaysia. Shahwahid et al. [52] used a convenience sampling design to select a sample from the local communities to identify their participation in tourism activities at Kampung Kuantan Firefly Park in Malaysia. Ratnasari et al. [53] have used purposive techniques to collect the survey data from the respondents through a study on halal tourism in Indonesia. Moreover, Khalique et al. [54] used the purposive survey technique in a study on tourism SMEs in Pakistan. For the present study, respondents were selected purposively from local communities in the study area and a questionnaire survey was conducted at the nearest Kampungs (villages) of each study location. The villages are selected purposively due to suitable access to the respondents. In total, 330 questionnaires were received from respondents with a response rate of 93 percent. Among them, 310 questionnaires were useable for the analysis. Among the respondents, 160 are from Kenyir and others from Sekayu.

### Questionnaire

This study used a structured questionnaire to determine the perception of the local communities. The questionnaire consists of two parts. The first part highlights the information to collect the demographic profile of respondents. The second part of the questionnaire intends to highlight the local communities’ perception of ecotourism development. This part includes eight statements on socio-economic aspects of ecotourism and uses a 5-point Likert-type scale ranging from 1 = strongly disagree, 2 = disagree, 3 = neither disagree nor agree, 4 = agree, 5 = strongly agree to the given statements. The questionnaire of this study can be found as supporting information (S1 Appendix).

### Data collection and analysis

In Sekayu Recreational Forest, the field survey has been conducted in July 2019 at the village Kampung Sekayu. Moreover, the field survey in Lake Kenyir has been completed by September 2019 from the two villages namely Kampung Basung and Kampung Pasir Dula. Primary data have been collected by a group of trained enumerators. For data analysis purposes, the study uses sophisticated factor analysis software FACTOR 8.10 to extract the latent factors attributed to the observed variables. LISREL 8.80 is used to perform the path analysis of the hypothesized structured equation of the latent factors and to obtain the fitness test of the confirmatory model.

### Methods

A two-step analysis has been carried out with the data to assess the local communities’ perception of ecotourism and its socio-economic impacts. First, exploratory factor analysis is done to observe the underlying constructs measuring the residents’ perception of tourism impacts. The obtained impact attributes were then examined by using the reliability estimates [55] and the root means square of residuals (RMR). After exploratory factor analysis, the study used Structural Equation Model (SEM) to determine the socioeconomic impacts of ecotourism on the livelihoods of local communities. A theoretical model was proposed for SEM and tested with respective path coefficients to examine the relationship between perceived tourism impacts and communities’ perceptions. SEM was developed by Joereskog [56] and his colleague as an efficient tool to deal with errors in variables. Ali et al. [57] argued that higher advantages are remaining for applying the SEM technique in tourism research. Islam et al. [58] (2020) have used SEM to test the validity of hypotheses regarding socio-economic tourism impacts in Bangladesh. Vukovic et al. [59] have analyzed communities’ perceptions of the impact of tourism development in rural areas through SEM. The study of Fakfare and Wattanacharoensil [60] studied tourism impacts on the community market in Thailand and used SEM to investigate the perceived well-being of communities.

Since the data file consists of a set of ordinal responses and the univariate distributions of ordinal items are asymmetric or with an excess of kurtosis, a factor analysis using Polychoric correlation is considered [61]. The appropriateness of factor analysis of the above correlation matrix obtained from the raw data was determined by examining the Kaiser-Meyer-Olkin (KMO) measure of sampling adequacy and Bartlett’s test of sphericity. A value of 0.60 or above for KMO statistics indicates that the data are adequate for exploratory factor analysis [62]. At the same time, significant Bartlett’s test of sphericity is also required. The optimal implementation of parallel analysis (PA) [63] is used for determining the number of dimensions to attain in the exploratory factor analysis. Factors are then extracted by using minimum rank factor analysis (MRFA) [64] and ‘PROMIN’ technique [76] is used for factor rotation to achieve factor simplicity. The reliability of the rotated factors is determined by respective reliability estimates [55] and the simplicity indices(S) by Bentler [65] and loading the simplicity index (LS) by Lorenzo-Seva [66] explains the factor simplicity to optimize the factor interpretation. Both the indices range from zero to one in which the greater value in the simplicity index(S) represents simpler and more interpretable factor solutions and the lower value of the loading simplicity (LS) index represents a maximum complexity [66]. Finally, the exploratory factor analysis model has verified with the value of the root mean square of residuals (RMR) is lower than the expected mean value of RMR for an acceptable model of 0.1060 [67].

SEM is used to investigate interrelationships between two types of variables: observed and latent. Observed variables can be directly measured by a researcher and latent variables are of interest to a researcher, but they are not directly observable. To obtain the covariance-based SEM converge attributes that have factor loading lower than the absolute value of 0.30 were eliminated from the analysis. At the individual path level, SEM estimates item loadings and measurement error along with their respective t-values. Construct reliability can be derived from the above statistics that should be above 0.70 [68]. The t-values associated with the path coefficients should be significant to support the hypothesized paths. The squared multiple correlations (SMC) of each of the exogenous latent constructs represented the explained variance of each latent construct [69]. An insignificant chi-square value identifies the good fit of the entire model [70]. The overall model fit indices are described in Table 2.

**Table 2:** The overall model fit indices of SEM.

Indices	Expected values	References
Chi-square Test	Low value is desired	[71]
Root Mean Square Residual (RMR)	below 0.05	[72, 73]
Root Mean Square Error of Approximation (RMSEA)	Less than .05	[70]
Goodness-of-fit Index (GFI)	Above 0.90	[72, 73]
Adjusted Goodness-of-fit Index (AGFI)	Above 0.90	[72, 73]
Incremental Fit Index (IFI)	Above 0.90	[74]
Normed Fit Index (NFI)	Above 0.90	[68, 72]
Relative Fit Index (RFI)	Ranges from zero (poor fit or no fit at all) to 1.0 (perfect fit).	[71]
Comparative Fit Index (CFI)	Above 0.90	[74]
Parsimonious Normed Fit Index (PNFI)	Higher values of the PNFI are better.	[75]
Parsimonious goodness-of-fit Index (PGFI)	Equal or larger than .50	[75]
Critical N (CN)	An excess of 200 is indicative and adequately represents the sample data.	[71]

In the present study, eight observed variables have been used to analyze the socio-economic impacts. The variables are the prices of goods, creating employment opportunities for residents, homestay accommodation opportunities, negative impacts of tourism, respect for cultural tradition, respect for religious tradition, degradation of natural resources and environment, and community participation in tourism development (Table 1). Data analysis has been carried out in two different steps. First, the positive and negative socio-economic impacts of each observed variable were observed by Factor Analysis. Based on the results, some hypotheses were proposed to examine the relationship between local communities’ perceptions of ecotourism impacts. In the second step, SEM is used to examine the relationship between observed variables with the latent variables and local community perceptions.

## Results

The demographic profile analysis revealed that most of the respondents from the local communities are male, married, and have primary and secondary level education. All the respondents are of Malay ethnicity and the majority of them are self-employed (52.5%). The self-employed respondents are farmers, cattlemen, rubber planters, gardeners, tour guides, and cleaners. The sample also includes some businessmen (35.5%) who are engaged in food and drink shops, traditional goods and handicrafts shops, grocery shops, homestay operators, tour operators, and other tourism services. This scenario indicated that most of the respondents are family people and are directly engaged in ecotourism activities in this area. Their educational background showed that they do have not enough opportunities to change their occupation.

In the structured questionnaire, respondents from local communities were asked about their perception of ecotourism impact with eight different questions. Responses were organized in a ‘Likert scale’ of measurement from strongly disagree to strongly agree to represent their perception accordingly. The descriptive characteristics of the selected variables are presented in Table 3.

**Table 3:** Univariate descriptives of selected variables (Sample size, n = 310).

Variables	Mean (95% Confidence Interval)	Variance	Skewness	Kurtosis
Increases prices of goods (PRICE)	2.87 (2.55, 3.18)	1.338	-0.041	-1.505
Employment opportunities for local residents (JOB)	4.00 (3.90, 4.10)	0.133	-2.769	19.753
Helpful for home stay accommodation (HOMESTAY)	3.99 (3.83, 4.14)	0.322	-1.846	6.492
Ecotourism has an overall negative impact (NEGIMP)	2.10 (1.86, 2.34)	0.757	1.135	0.813
Respectful to cultural tradition (CULTRAD)	3.99 (3.92, 4.05)	0.055	-5.883	58.040
Respectful to religious tradition (RELTRAD)	3.97 (3.86, 4.08)	0.166	-3.254	16.800
Degradation natural resources (NATUENV)	2.08 (1.86, 2.30)	0.650	1.273	1.539
Participation for tourism development (TOURDEV)	3.99 (3.77, 4.03)	0.223	-4.778	21.742

As the results in Table 3 represent the ordinal response in the Likert Scale that is asymmetric and with an excess of kurtosis, the polychoric correlation is used for factor analysis [61].

Before starting the dimensionality of the data matrix, the adequacy of the correlation matrix was checked, and a significant Bartlett’s test (Statistic value = 133.3 with d.f. = 28, p<0.001) along with a moderate Kaiser-Meyer-Olkin (KMO) index (KMO statistic = 0.60) are observed. That verifies the sample adequacy for obtaining the significant dimensionality from the correlation matrix. Finally, the sample data matrix shows the existence of two-dimensional latent variables information in the factor rotation using ‘PROMIN’ rotation method [76]. The detailed result of factor analysis is given in Table 4.

**Table 4:** Results of factor analysis.

Variable	Factor-1 (Negative Socio-economic Impact)	Factor-2 (Positive Socio-economic Impact)
Increases prices of goods (PRICE)	0.017	0.148
Employment opportunities for local residents (JOB)	-0.106	**0.854**
Helpful for home stay accommodation (HOMESTAY)	-0.168	**0.648**
Ecotourism has an overall negative impact (NEGIMP)	**0.972**	-0.037
Respectful to cultural tradition (CULTRAD)	-0.244	0.099
Respectful to religious tradition (RELTRAD)	**-0.390**	0.184
Degradation natural resources (NATUENV)	**0.688**	-0.080
Participation for tourism development (TOURDEV)	0.020	**0.652**
**Related estimates**		
Eigen value	1.650	1.627
Proportion of variance explained	0.377	0.372
Reliability estimates	0.953	0.810
**Indices for factor simplicity**		
**Bentler’s simplicity index (S)**	0.99976	
**Lorenzo-Seva simplicity index (LS)**	0.61367	
**Root mean Squared Residual (RMR)**	0.0585	

*The bold values indicating the factor ladings greater than the considered 0.30 absolute threshold

Table 4 shows the result of factor analysis of the statements of local peoples’ perceptions regarding socio-economic impact. The result indicates that latent factor-1 (negative socio-economic impact) is highly positively associated with the respondents’ perception that ecotourism has an overall negative impact and also makes degradation of natural resources. At the same time, this factor is slightly associated with the perception of the break-up of religious traditions in the locality. We may consider this factor (factor-1) as a negative socio-economic impact on ecotourism. The latent factor-2 (positive socio-economic impact) can be observed with a higher association with the respondents’ perception of local employment opportunities, their participation in tourism development, and a positive response associated with homestay accommodation increment. All these responses can be defined as a positive impact on ecotourism experienced by the residents. Here, two variables ‘ecotourism increases prices of goods’ and ‘ecotourism respectful to cultural tradition’ are excluded for their lower factor loading (<0.30) in the analysis. The reliability estimates are higher than the suggested 0.70 thresholds for both factors. The greater value (0.99967) of Bentler’s simplicity index (S) along with a moderate value of the Lorenzo-Seva index (0.6136) implies simplicity and less complexity in the factor rotation. The obtained RMSR value (0.0585) is much less than the expected mean value of RMSR = 0.1060 for an acceptable model (Kelly’s criterion).

Based on the above result, the conceptual and empirical perspectives from tourism literature, and the results obtained from the exploratory analysis, we may propose some hypotheses to examine the relationship between local communities’ perception of ecotourism impacts. The proposed hypotheses are:

**Hypothesis 1**: A direct positive relationship exists between the positive impact of ecotourism and employment opportunities for local communities.
**Hypothesis 2**: A direct positive relationship exists between the positive impact of ecotourism and the opportunity for homestay accommodation for local communities.
**Hypothesis 3**: A direct positive relationship exists between the positive impact of ecotourism and local communities participation in tourism development.
**Hypothesis 4**: A direct positive relationship exists between the negative impact of ecotourism and local communities’ overall negative attitude towards ecotourism.
**Hypothesis 5**: A direct positive relationship exists between the negative impact of ecotourism and local communities’ perception that ecotourism development is degrading natural resources.
**Hypothesis 6**: A direct negative relationship exists between the negative impact of ecotourism and local communities’ perception that ecotourism is disregarding religious tradition.

**Fig 1:**
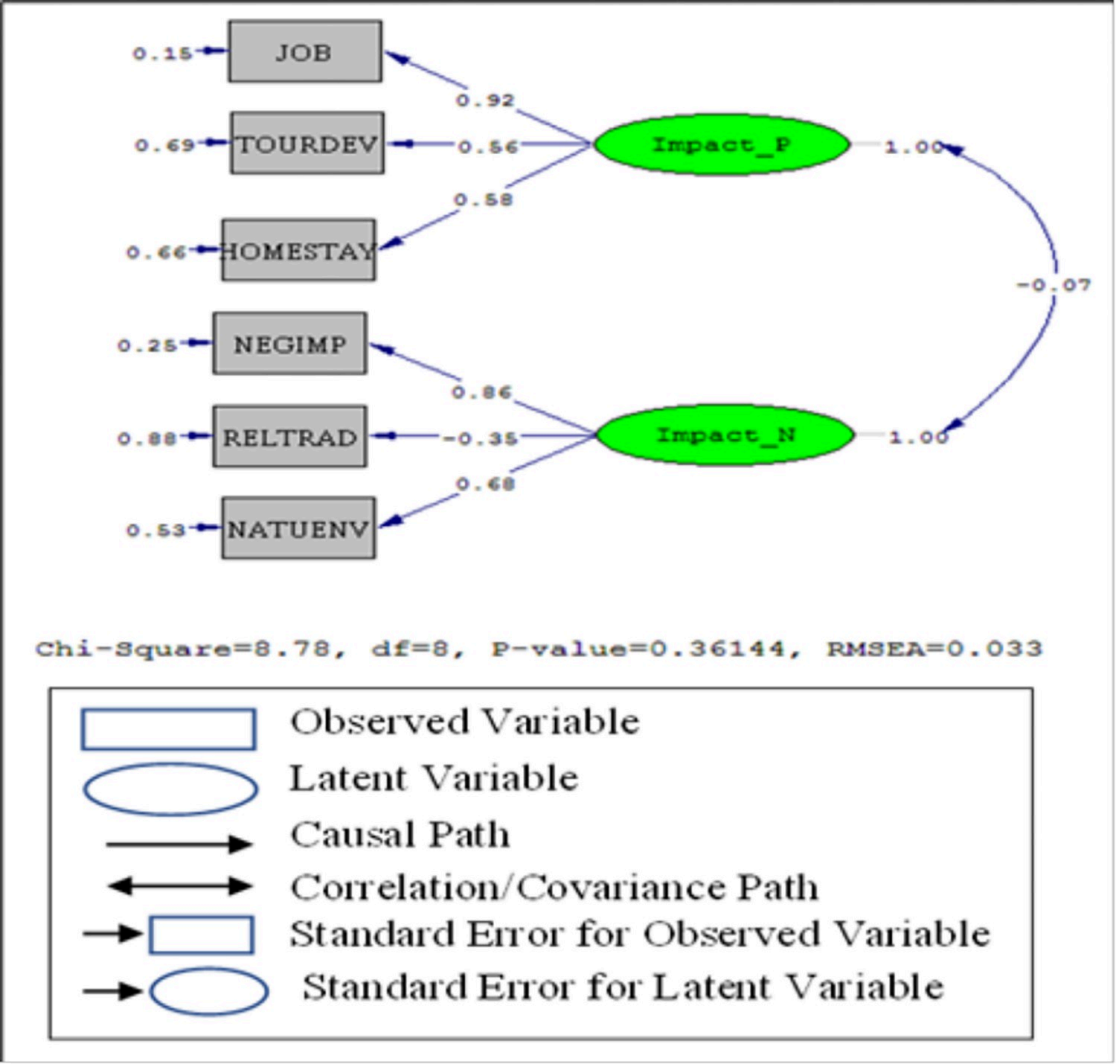
Structural equation model parameters for the impacts of ecotourism development (LISREL outputs).

To determine these causal relationships, six path coefficients were estimated considering structural equation modeling (SEM), and the result is shown in Fig 1. In the LISREL output for the path analysis, all the standard loading indices found to be significant accept the loading between the latent variables (-0.07), which is almost zero. Therefore, no significant path exists between the latent variables (Fig 1).

Table 5 represents the values of model fit indices in SEM. An insignificant chi-square value along with an RMSEA less than 0.05 implies the entire goodness of fit of the postulated structured model. The overall fitness indices GFI (0.97>0.90); AGFI (0.92>0.90) and RMR (0.023<0.05) are within the satisfactory threshold limits. Moreover, other fit indices IFI (0.99>0.90); CFI (0.99>0.90); RFI (0.85) and CN (205.41) are also within the satisfactory limits. Furthermore, the values of indices NFI (0.92) are above the 0.90 thresholds. The higher value of PNFI (0.49) also indicates a satisfactory level for model fit. An analysis of estimated standard path coefficients in the proposed structural model identifies the significance, strength, and direction of each hypothesized relationship. All the directions of postulated paths in the proposed theoretical model are statistically significant in their direction predicted at the 0.05 level. Thus, the result of the structural equation modeling revealed the nomological validity of two dimensions of impacts towards ecotourism that were observed in the exploratory factor analysis.

**Table 5:** Values of model fit indices in SEM to the data.

Indices	Expected values	Model values
Chi-square Test	Low value is desired	8.78 (p = 0.36)
Root Mean Square Residual (RMR)	below 0.05	0.023
Root Mean Square Error of Approximation (RMSEA)	Less than .05	0.03
Goodness-of-fit Index (GFI)	Above 0.90	0.97
Adjusted Goodness-of-fit Index (AGFI)	Above 0.90	0.92
Incremental Fit Index (IFI)	Above 0.90	0.99
Normed Fit Index (NFI)	Above 0.90	0.92
Relative Fit Index (RFI)	Ranges from ‘zero’ (poor fit or no fit at all) to ‘1.0’ (perfect fit).	0.85
Comparative Fit Index (CFI)	Above 0.90	0.99
Parsimonious Normed Fit Index (PNFI)	Higher values of the PNFI are better.	0.49
Parsimonious goodness-of-fit Index (PGFI)	Equal or larger than .50	0.57
Critical N (CN)	An excess of 200 is indicative and adequately represents the sample data.	205.41

The above analysis reveals that local communities are showing positive perceptions of the positive impacts of ecotourism development. They have expressed positive perceptions of employment opportunities, homestay accommodations, and community participation of local people in ecotourism development in the study areas. They have expressed negative perceptions of the overall negative impacts of ecotourism and the degradation of natural resources and the environment by ecotourism development. Again, the study shows that a negative relationship exists between the negative impact of ecotourism and local people’s perception that ecotourism is degrading religious tradition.

## Discussion

The study shows a direct positive relationship between the positive impact of ecotourism on local communities with employment opportunities and is helpful for homestay accommodation. Several studies [5, 44] represented that economic benefits are the main considering elements required by local people for ecotourism development in an area. Ecotourism has ensured economic benefits for the locals in Ethiopia [5] and Uttarakhand in India [44]. Previous researchers also reported that the local residents feel tourism as employment creating opportunities [16, 17] and bringing new business opportunities [20] for local communities. Again, ecotourism helps the homestay accommodations in the rural areas in Nepal [15, 17] which supports the outcomes of this study. The study reveals a direct positive relationship exists between the positive impact of ecotourism and local communities participation in tourism development. The study finding demonstrates that local communities have experienced social and economic betterment after ecotourism development in the study areas. The study findings of [10, 11] show that ecotourism gives participation opportunities to the local people in planning and decision-making respectively.

Based on the perception of local communities, the study identified that ecotourism development needs to ensure benefits for the locals in terms of employment opportunities, homestay accommodation, and community participation in decision-making. The studies [32, 36] in Malaysia supported this finding that ecotourism development creates employment opportunities for the locals. The studies [37, 38] on homestays in Malaysia revealed that ecotourism development is helpful for homestay accommodation which ensures local communities’ benefits. Moreover, several studies in Malaysia [23, 52] emphasized local community participation in ecotourism development.

The study identified a direct relationship between the negative impact of ecotourism and local communities’ perception that ecotourism development is degrading natural resources. The study indicates that a relationship exists between the negative impact of ecotourism and local communities’ overall negative perception of ecotourism development. This is supported by the statement in some previous literature- ecotourism can negatively influence the natural resources and environment in mountain areas of the USA [21], cause cultural commercialization in Ghana [6], and create social and cultural conflicts in the community due to socio-economic differences and economic benefits in Ethiopia [5]. The present study shows local communities express negative perceptions of the degradation of natural resources and the environment. The study of [18] on Turkey and [21] on the USA emphasized environmental advantages and environmental conservation respectively to improve the livelihoods of local communities. The study found a direct negative relationship exists between the negative impact of ecotourism and local people’s perception that ecotourism is degrading religious tradition. The findings support the research outputs of [22] at Sauraha in Nepal where tourism development has influenced the local communities to change their religious beliefs.

The study identifies that local communities are not supporting some shortcomings of ecotourism development like negative impacts of ecotourism, degradation of natural resources and environment, and degrading religious tradition. The study of [28] on Malaysia supported the study finding that local communities do not support the negative impacts due to ecotourism development. The study finding of [32] on Terengganu is also similar where local communities showed negative perception towards ecotourism development due to degradation of natural resources. Furthermore, several studies [39, 41] on Malaysia also emphasized on conservation of natural resources to ensure benefits for local communities. Again, the study of [33] on Terengganu identified that respect for religious tradition increases the positive perception of local communities toward ecotourism development.

## Conclusion

The study is providing a guideline for ecotourism development in an area to consider the perception of local communities to ensure their well-being. It is highlighting the components for gathering socio-economic benefits of local communities. The study shows that employment opportunities, homestay accommodations, and community participation scopes are experienced as positive socio-economic impacts on ecotourism development. This is indicating that the local communities think that ecotourism development creates employment and homestay accommodation business opportunities for local people. Furthermore, they feel ecotourism development also ensures their active participation in decision making, planning, and management of tourism activities. Thus, ecotourism is showing positive impacts on the livelihood of local communities and ensuring their socio-economic benefits.

The study revealed that overall negative impacts and degradation of natural resources have negative socio-economic impacts on ecotourism development. Through the study finding, local communities believe that ecotourism is slightly associated with the break-up of religious traditions. These impacts create a negative influence on ecotourism development and reduce the well-being of local communities. It also revealed that the respondents are impartial to the impact of ecotourism development on price increments of goods and considerate of the cultural traditions.

Ecotourism development in the study areas must consider the negative socio-economic impacts and try to ensure the well beings of local communities. So, these negative impacts should be minimized in study areas for sustainable ecotourism development and to ensure benefits for the local communities. This study recommends a thorough study of how the local people participate and get benefits from ecotourism development in the study area.

The study is providing necessary policy components for ecotourism development in the other parts of Malaysia as well as the global context. The study revealed that community benefits are a must for gathering support from the locals for ecotourism development. Again, local communities are not supporting ecotourism development if gathering negative outcomes on their livelihood. The study suggested that several positive outcomes like providing employment opportunities, promoting local accommodations, and ensuring community participation are must for gathering benefits for locals through ecotourism development. Moreover, ecotourism development must be free from some shortcomings such as creating negative impacts, degradation of natural resources and environment, and degrading religious beliefs and traditions. For further research, it will be a scope to identify related problems by ecotourism development and necessary solutions in this regard.

## Supporting information

S1 Appendix Questionnaire for local community. (DOCX)
