# Supplementary material for: A Structural Equation Model (SEM) for the socio-economic impacts of ecotourism development in Malaysia
Source: PLoS One. 2022 Aug 29;17(8):e0273294. doi: 10.1371/journal.pone.0273294 (PMC9423673; doi:10.1371/journal.pone.0273294)
Supplement: S1 Appendix — (DOCX) [file pone.0273294.s001.docx]

**S1-APPENDIX**

**Questionnaire for Local Community**

**Tourist site: a) Sekayu b) Lake kenyir [Please (√) and give comments]**

1. Occupation: ………………….
2. Sex: a) Male b) Female
3. Marital Status: a) Married b) Single
4. Education:
5. Primary education
6. Secondary / High School

b) College / Diploma

c) University education

6. Ethnic groups

1. Malay
2. Chinese
3. Indian
4. Others……….

**Socioeconomic Impacts of ecotourism**

| Statements | Strongly disagree | Disagree | Neutral | Agree | Strongly agree |
| --- | --- | --- | --- | --- | --- |
| Ecotourism increases the prices of goods |  |  |  |  |  |
| Ecotourism influences employment opportunities for residents |  |  |  |  |  |
| Ecotourism helpful for homestay accommodation |  |  |  |  |  |
| Ecotourism creates negative impacts |  |  |  |  |  |
| Ecotourism activities is respectful of cultural tradition |  |  |  |  |  |
| Ecotourism activities is respectful of the religious tradition |  |  |  |  |  |
| Ecotourism creates degradation of natural resources and the environment |  |  |  |  |  |
| Ecotourism ensures community participation in tourism development |  |  |  |  |  |
